# From Design to Bioactivity: 2‐Benzoylpyridine 4‐(bicyclo[2.2.1]hept‐2‐yl)thiosemicarbazone and Its 3*d* Metal Coordination Compounds

**DOI:** 10.1002/open.202500590

**Published:** 2026-03-29

**Authors:** Ianina Graur, Vasilii Graur, Elena Melnic, Carolina Lozan‐Tirsu, Greta Balan, Victor Tsapkov, Aurelian Gulea

**Affiliations:** ^1^ Laboratory of Advanced Materials in Biopharmaceutics and Technics Institute of Chemistry Moldova State University Chisinau Republic of Moldova; ^2^ Laboratory of Physical Methods for Studying Solids “Tadeusz Malinowski” Institute of Applied Physics Moldova State University Chisinau Republic of Moldova; ^3^ Department of Preventive Medicine State University of Medicine and Pharmacy “Nicolae Testemitanu” Chisinau Republic of Moldova

**Keywords:** 3*d* metal complexes, antibacterial activity, antifungal activity, thiosemicarbazone

## Abstract

The new 2‐benzoylpyridine 4‐(bicyclo[2.2.1]hept‐2‐yl)thiosemicarbazone (**HL**) and its copper(II), nickel(II), cobalt(III), and iron(III) coordination compounds [Cu(L)Cl] (**1**), [Cu(L)NO_3_] (**2**), {[Cu(L)(Cl_2_CHCOO)]}_*n*_ (**3**), [Ni(HL)_2_](NO_3_)_2_ (**4**), [Fe(L)_2_]NO_3_ (**5**), [Co(L)_2_]NO_3_ (**6**) were obtained. The thiosemicarbazone **HL** was studied using ^1^H, ^13^C NMR, and FTIR spectroscopy. All the obtained 3*d* metal coordination compounds were characterized by elemental analysis and FTIR spectroscopy. The single‐crystal X‐ray diffraction analysis was used to determine the crystal structures of **HL** and complex **3**. The study of antibacterial activity revealed that coordination of the thiosemicarbazone **HL** to the metal ions leads to an increase in activity. Whereas in the case of antifungal activity, the effect is the opposite—the activity either remains at the same level or decreases. Most of the complexes exhibited activity that surpassed the results obtained for the standard drugs Furacillinum, Nystatin, and Fluconazole.

## 1 Introduction

Since the discovery of their cytotoxic and anticancer properties, thiosemicarbazones have attracted considerable research interest [1]. Transition‐metal complexes derived from thiosemicarbazones are especially appealing due to the straightforward synthesis of structurally diverse compounds and their wide spectrum of biological effects [2]. These ligands and their complexes have been investigated as potential antiviral [3], antimicrobial [4, 5, 6], anti‐inflammatory [7], antimalarial [8], antileukemic [9], and anticancer [10] agents, as well as for their antioxidant capacity [11] and ability to bind DNA [12]. Owing to these activities, the thiosemicarbazone fragment is regarded as an important pharmacophore in drug design.

The pronounced chelating ability of thiosemicarbazones toward transition metals such as Fe, Ni, Cu, and Zn, together with their redox properties, underlies much of their pharmacological potential [13, 14, 15, 16]. Coordination to metal ions often alters their biological profile—for instance, by modifying lipophilicity, which influences cellular uptake and may help mitigate side effects. Additionally, metal complexation can lead to novel bioactivities not observed in the uncoordinated thiosemicarbazone ligands. Several reports indicate that introducing bulky substituents at the *N*(4) position of the thiosemicarbazone fragment can significantly improve its biological activity [17, 18]. One example is a series of thiosemicarbazones containing an *N*(4)‐azabicyclo [3.2.2]nonane moiety derived from 3‐acylpyridazines, 4‐acetylpyrimidines, and 2‐acetylpyrazines, which were synthesized as potential anticancer agents. They show promising activity against human acute lymphoblastic leukemia CCRF‐CEM cells with IC_50_ = 0.05−0.77 μM and colon adenocarcinoma HT‐29 cells with IC_50_ = 0.05−0.77 μM [19].

2‐Benzoylpyridine is a recently identified antimicrobial agent with strong activity, acting primarily through disruption of the bacterial membrane. Interacting with the membrane, it induces leakage of ions and small molecules, leading to disturbances in the redox balance of the cell. In addition to its antimicrobial properties, this compound has demonstrated notable cytotoxic effects against human colon adenocarcinoma cells in vitro. Its molecular structure allows for intermolecular hydrogen bonding through its coordination geometry, a feature that may contribute to its pronounced antimicrobial effectiveness. 2‐Benzoylpyridine thiosemicarbazones are less frequently reported in the literature compared to other derivatives containing a pyridine ring, such as 2‐acetylpyridine and 2‐formylpyridine thiosemicarbazones. Nevertheless, heterocyclic thiosemicarbazones have long been recognized as potential anticancer agents. 2‐Benzoylpyridine thiosemicarbazones and their metal coordination compounds exhibit cytotoxic activity towards various cancer cell lines, such as lung cancer A549 [20], human melanoma UACC‐62, human renal carcinoma TK‐10, human breast cancer MFC‐7 [21], and erythroleukemia cancer K562 [22, 23]. In addition, these compounds exhibit antibacterial [24, 25, 26] and antifungal [27, 28] activities, and in most cases, the coordination of 2‐benzoylpyridine thiosemicarbazone to the metal atoms leads to an enhancement of their biological activity.

Previously, we synthesized 2‐benzoylpyridine 4‐allylthiosemicarbazone and its coordination compounds with copper(II), nickel(II), zinc(II), and iron(III), and investigated their antibacterial, antifungal, and antioxidant activities [29]. In another study, we also reported 2‐acetylpyridine 4‐(bicyclo [2.2.1]hept‐2‐yl)thiosemicarbazone and its copper(II) coordination compounds, as well as their antibacterial activity against Gram‐positive and Gram‐negative microorganisms, which demonstrated interesting results [30]. Therefore, it is of interest to investigate how the combination of two structural fragments from previously obtained compounds affects the chemical and biological properties of the resulting substances.

**FIGURE 1:**
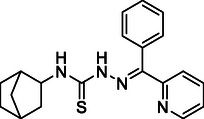
The structural formula of **HL**.

Based on the above, the aim of this work is the synthesis, characterization, and investigation of the antibacterial, antifungal, and anticancer activities of 2‐benzoylpyridine 4‐(bicyclo [2.2.1]hept‐2‐yl)thiosemicarbazone (**HL**) (Figure 1) and its copper(II), nickel(II), iron(III), and cobalt(III) coordination compounds.

## 2 Results and Discussion

**SCHEME 1:**
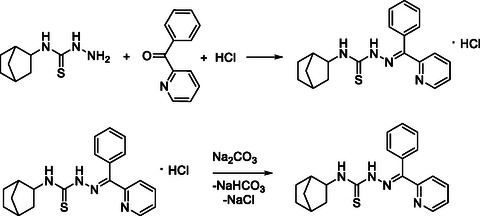
Synthesis of 2‐benzoylpyridine 4‐(bicyclo [2.2.1]hept‐2‐yl)thiosemicarbazone (**HL**).

In this work, a new 2‐benzoylpyridine 4‐(bicyclo [2.2.1]hept‐2‐yl)thiosemicarbazone (**HL**) was obtained. Its synthesis was carried out in two steps. In the first stage, 4‐(bicyclo [2.2.1]hept‐2‐yl)thiosemicarbazide, 2‐benzoylpyridine, and concentrated hydrochloric acid were mixed in ethanol in a 1:1:1 molar ratio and stirred under heating. In the second stage, the resulting hydrochloride salt was dissolved in ethanol and neutralized using an aqueous solution of Na_2_CO_3_ (Scheme 1).

The ^1^H and ^13^C NMR spectroscopy (Figures S1 and S2) was used to confirm the structure of thiosemicarbazone (**HL**). The ^1^H NMR spectrum contains characteristic peaks of the 2‐benzoylpyridine fragment at 7.76, 7.60, 7.45, 7.37, and 7.27 ppm as well as multiplets at 2.45, 2.32, and 2.0–1.1 ppm which correspond to the bicyclic fragment.

**SCHEME 2:**
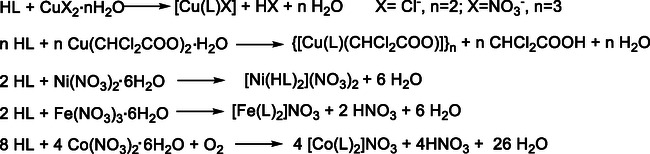
Synthesis of the coordination compounds **1**–**6**.

Six new Cu(II), Ni(II), Fe(III), and Co(III) coordination compounds with **HL** were synthesized (Scheme 2). Copper(II) complexes **1**‐**3** were obtained by reaction between the thiosemicarbazone (**HL**) and copper(II) salts in ethanol at a 1:1 molar ratio. Nickel(II) (**4**), cobalt(III) (**6**), and iron(III) (**5**) complexes were synthesized through the interaction of the corresponding metal salts with **HL** in ethanol at a 1:2 molar ratio. The resulted coordination compounds have the following composition: [Cu(L)Cl] (**1**), [Cu(L)NO_3_] (**2**), [Cu(L)CHCl_2_COO]_*n*_ (**3**), [Ni(HL)_2_](NO_3_)_2_ (**4**), [Fe(L)_2_]NO_3_ (**5**), [Co(L)_2_]NO_3_ (**6**). All the obtained compounds are soluble in organic solvents such as MeOH, EtOH, DMF, and DMSO. The study of the molar conductivity of the obtained complexes **1–** **3**, **5–** **6** showed that these compounds are 1:1 type of electrolytes, with values in the range of 46–80 Ω^−1^·cm^2^·mol^−1^. Only in the case of nickel complex **5**, the molar conductivity value is 134 Ω^−1^·cm^2^·mol^−1^, indicating that this complex is a 1:2 type of electrolyte.

The FTIR spectra of complexes **1–6** (Figures S3–S9) were analyzed in comparison with those of the free thiosemicarbazone **HL** to identify spectral changes associated with metal coordination. The data indicate that **HL** coordinates through its nitrogen and sulfur donor atoms. For complexes **1–3** and **5–6**, the ν(NH) stretching band shifts by 47–74 cm^−1^ toward higher frequencies, while the second ν(NH) band disappears entirely, suggesting deprotonation of the ligand during metal binding. In contrast, both ν(NH) absorption bands persist in the spectrum of the Ni(II) complex (**4**), implying that in this case thiosemicarbazone remains in its neutral form. Furthermore, new ν(C = N) bands appear in the spectra of complexes **1–3** and **5–6**, while the characteristic ν(C = S) band of the free ligand vanishes, confirming coordination through the thiol form of the ligand. The new ν(C–S) absorption bands emerge in the 743–748 cm^−1^ region for these complexes. However, in complex **4**, the ν(C = S) absorption band remains visible, consistent with the retention of the thione form of the ligand.

The UV‐Vis spectra of **HL** and complexes **1**‐**6** were compared to confirm the complexation process. It was observed that the spectra of copper(II) complexes **1–** **3** are identical. This suggests that upon dissolution, the coordination environment changes: compounds **1–** **3** undergo dissociation, the acid residue anions are removed from the inner coordination sphere, and complex cations are generated consisting of the central copper ion coordinated to the thiosemicarbazone ligand **HL**.

**FIGURE 2:**
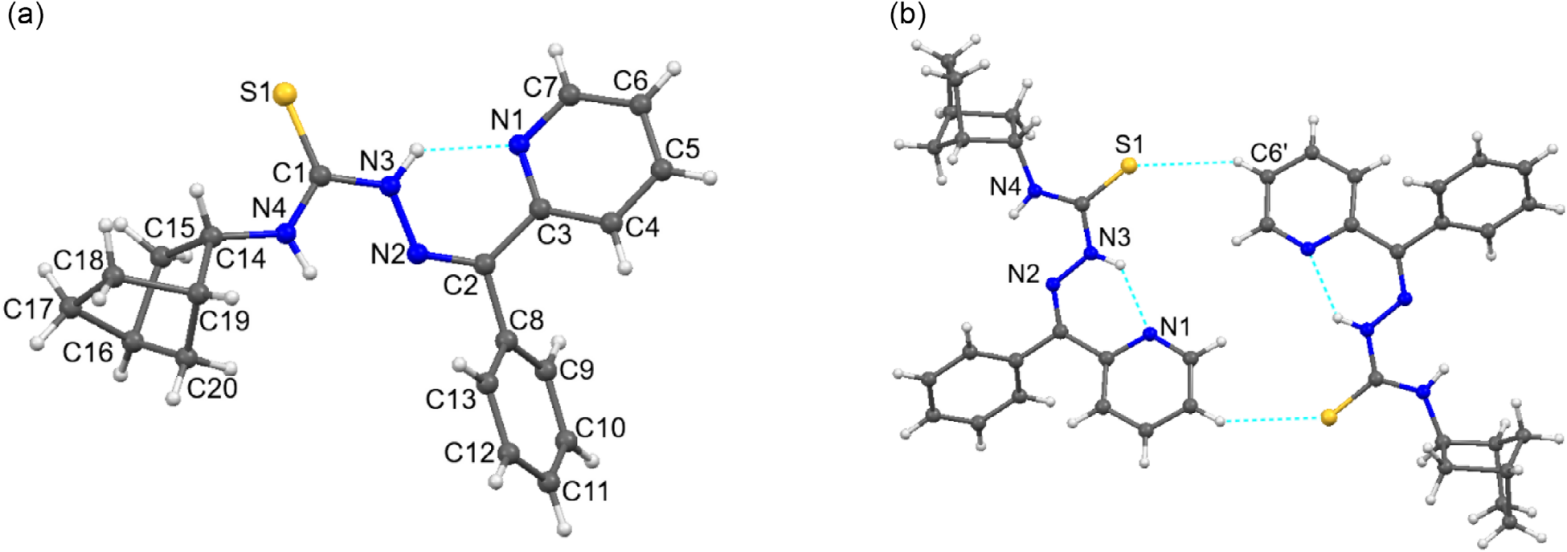
Molecular structure of compound **HL** with atom numbering scheme (a); view of the supramolecular dimer of **HL** (b).

Single crystals of **HL** and compound **3** were obtained from ethanol by recrystallization. Single‐crystal X‐ray diffraction analysis revealed that **HL** and copper(II) coordination compound **3** crystallize in the monoclinic *P*2_1_/*n* and *P*2_1_/*c* space groups, respectively (Table 1). The crystal structure of **HL** shows that the nearly planar within 0.098 Å thiosemicarbazone moiety adopts an E configuration. The nitrogen atom (N1) of the pyridine moiety forms an intramolecular hydrogen bond with the nitrogen atom (N3) of the thiosemicarbazone fragment with an N3–H···N1 distance of 2.691(3) Å (Table S2), stabilizing the orientation of the pyridine ring (Figure 2a). The mean plane of the thiosemicarbazone moiety forms dihedral angles of 26.16° and 44.88° with the planes of the pyridine and phenyl rings, respectively.

**TABLE 1:** Crystallographic data and structure refinement details for compounds HL and 3.

	HL	3
CCDC	2493073	2493074
Empirical formula	C_20_H_22_N_4_S	C_22_H_22_Cl_2_Cu_1_N_4_O_2_S
Formula weight	350.47	540.93
Crystal system	Monoclinic	Monoclinic
Space group	*P*2_1_/*n*	*P*2_1_/*c*
Z	4	4
*a* (Å)	6.0721 (3)	14.2885 (7)
*b* (Å)	18.5106 (11)	18.4380 (11)
*c* (Å)	16.7437 (10)	9.5574 (5)
α (deg)	90	90
β (deg)	92.964 (5)	106.981 (5)
γ (deg)	90	90
*V* (Å^3^)	1879.4 (2)	2408.1 (2)
*D*_c_ (g/cm^–3^)	1.239	1.492
µ_Mo_ (mm^−1^)	0.182	1.242
*F*(000)	744	1108
Crystal size (mm^3^)	0.50 × 0.40 × 0.22	0.20 × 0.10 × 0.06
θ range (°)	3.284 to 25.048	2.981 to 25.049
Index range	−5 ≤ *h* ≤ 7, −22 ≤ *k* ≤ 20, −19 ≤ *l* ≤ 19	−17 ≤ *h* ≤ 16, −12 ≤ *k* ≤ 21, −7 ≤ *l* ≤ 11
Reflections collected/unique	6784/3310 [*R*_int_ = 0.0210]	8974/4238 [*R*_int_ = 0.0726]
Reflections with, [*I* > 2σ(*I*)]	2363	2170
Parameters	263	292
GOF on F^2^	1.001	0.999
*R*_1_, w*R*_2_ [*I* > 2σ(*I*)]	0.0529, 0.1364	0.0714, 0.1148
*R*_1_, w*R*_2_ (all data)	0.0789, 0.1535	0.1499, 0.1431
Δρ_max_/Δρ_min_ (e·Å^−3^)	0.225/−0.191	0.558/−0.423

In the **HL** crystal structure, inversion‐related molecules are connected by intermolecular C–H···S hydrogen bonds (Table S2), forming a supramolecular dimer (Figure 2b).

The crystal structure of compound **3** reveals the formation of a one‐dimensional (1D) coordination polymer, {[Cu(L)(Cl_2_CHCOO)]}_*n*_. In this structure, the monodeprotonated (L^−^) undergoes a conformational change from the E to the Z compared with **HL**. The Cu(II) center coordinates to the ligand in the NNS chelating mode, forming two fused coplanar five‐membered metallacycles CuSCNN and CuNCCN with a dihedral angle 2.48° between their mean planes.

**FIGURE 3:**
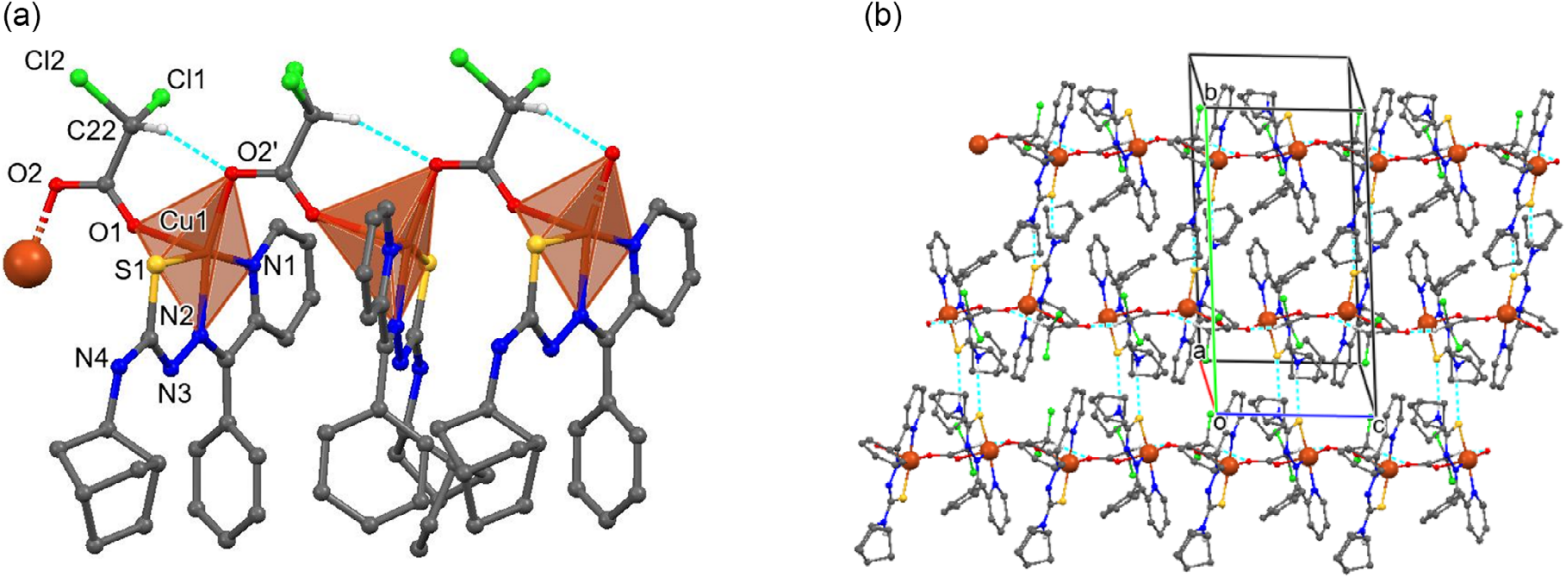
Fragment of the polymeric chain in the crystal structure of **3** with the partial atom numbering scheme (a). The supramolecular layer in **3** (b). Non‐functional H–atoms are omitted for clarity.

The CuL moieties link into a polymer chain via the bridging function of the dichloroacetate anion. The Cu(II) center is pentacoordinated and adopts a SN_2_O_2_ square‐pyramidal geometry (τ = 0.04) (Figure 3a). The bond distances in the coordination polyhedron Cu(1)–O(1), Cu(1)–N(1), Cu(1)–N(2), Cu(1)–S(1) and Cu(1)–O(2′) equal 2.320(4), 2.009(5), 1.958(4), 2.268(2), and 1.967(3) Å, respectively (Table S1). The Cu1 atom displaces from the mean basal plane of the base in the direction of the vertex of the pyramid by 0.1148 Å. The 1D polymeric chain is additionally stabilized by an intrachain C–H···O = 3.158(8) Å hydrogen bonds (Table S1). The Cu···Cu separation along the chain equals 4.825 Å. In the crystal, parallel coordination chains propagate along the *c* axis. The chains are interconnected by N(4)–H(4N)···S(1) hydrogen bonds (3.530(5) Å), forming in supramolecular layers parallel to (*bc*) plane.

The antibacterial and antifungal potential of the **HL** and complexes **1**–**6** was evaluated against Gram–positive bacteria (*S. aureus*, *B. cereus*), Gram–negative bacteria (*A. baumannii*, *E. coli*), and fungi (*C. albicans*). The obtained results in the form of minimum inhibitory/bactericidal/fungicidal concentrations are shown in Table 2.

**TABLE 2:** Antibacterial and antifungal activities of HL and complexes 1–6 as MIC/MBC/MFC values in μg mL^−1^.

Compound	*Staphylococcus aureus* ATCC 25 923		*Bacillus cereus* ATCC 11 778		*Acinetobacter baumannii* BAA‐747		*Escherichia coli* ATCC 25 922		*Candida albicans* ATCC 10 231	
	MIC	MBC	MIC	MBC	MIC	MBC	MIC	MBC	MIC	MFC
**HL**	—	—	—	—	—	—	—	—	0.98	0.98
**1**	0.24	0.49	0.49	0.49	31.25	125.0	500.0	—	0.24	1.95
**2**	0.98	1.95	0.24	0.24	7.8	15.6	500.0	—	0.98	0.98
**3**	1.95	1.95	0.98	0.98	62.5	125.0	—	—	0.24	1.95
**4**	—	—	500.0	—	250.0	500.0	500.0	500.0	125.0	—
**5**	7.8	15.6	7.8	7.8	500.0	—	—	—	250.0	500.0
**6**	1.95	3.9	0.49	1.95	250.0	250.0	500.0	500.0	15.6	31.3
Furacillinum	9.3	9.3	4.7	4.7	18.5	37.5	4.7	9.4	—	—
Nystatin	—	—	—	—	—	—	—	—	80	80
Fluconazole	—	—	—	—	—	—	—	—	15.6	31.3

Abbreviations: MIC, minimum inhibitory concentration; MBC, minimum bactericidal concentration; MFC, minimum fungicidal concentration; “–”, not active.

The synthesized thiosemicarbazone **HL** showed no activity against Gram‐positive or Gram‐negative microorganisms, exhibiting results only in antifungal testing. Its coordination to copper(II), nickel(II), iron(III), and cobalt(III) ions led to an increase in antibacterial activity and, in some cases, antifungal activity of the resulting complexes. The highest activity was observed against Gram‐positive microorganisms and fungi. Considering the results for the copper(II) complexes **1–** **3**, the influence of the counterion on biological activity can be observed. However, it is difficult to establish a general trend across all microorganisms since the dependence of activity on the counterion varies in each case. The results obtained for metal complexes derived from nitrate salts **2**, **4–** **6** allow the influence of the central metal ion on the activity of the complexes to be evaluated. For Gram‐positive microorganisms, the activity decreases in the following order: Cu^2+^ > Co^3+^ > Fe^3+^ > Ni^2+^.

The antifungal activity results showed that the thiosemicarbazone **HL** is less active than copper(II) complexes **1** and **3**, and its activity is comparable to that of complex **2**. All other complexes were less active, confirming that coordination of the ligand to a metal ion does not always enhance antifungal activity.

Standard drugs used in medicine were applied for comparison. Most of the synthesized complexes surpassed Furacillinum in activity against Gram‐positive microorganisms, and complex **2** also demonstrated higher activity against *Acinetobacter baumannii*. Nystatin and Fluconazole were used as standard antifungal agents, both of which showed lower activity than the thiosemicarbazone **HL** and its copper(II) and cobalt(III) complexes.

**FIGURE 4:**
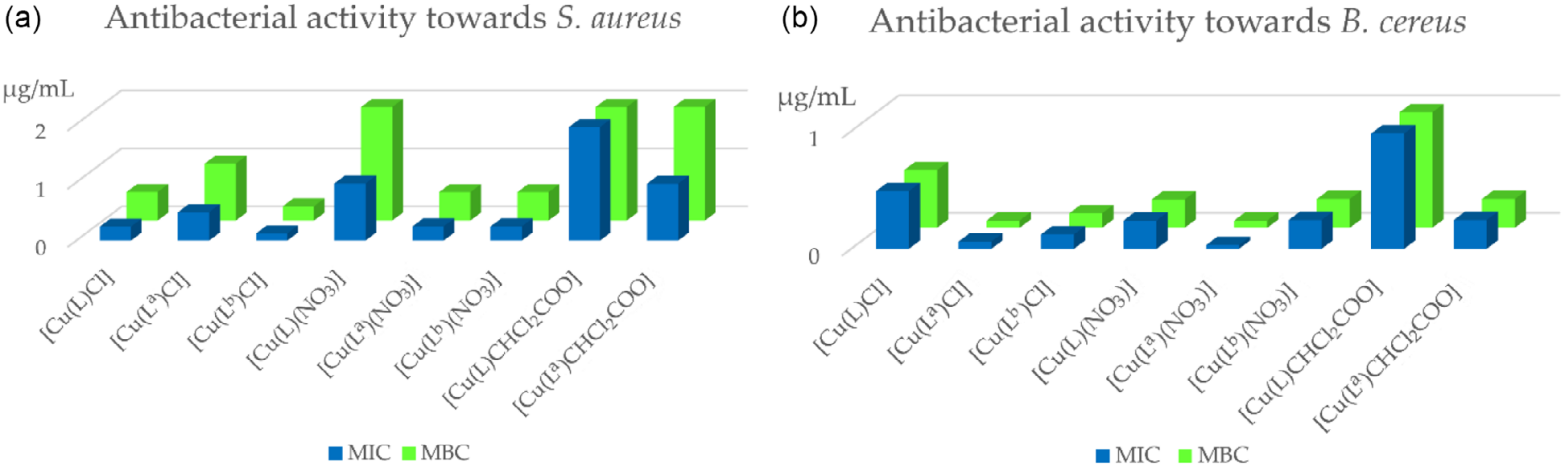
Comparison of the activity of studied copper(II) complexes with their analogs against *S. aureus* (a) and *B. cereus* (b).

The antibacterial activity results are evaluated not only by comparing the obtained compounds with each other, but also a comparison was made with previously synthesized and published compounds containing similar structural fragments: 2‐acetylpyridine 4‐(bicyclo [2.2.1]hept‐2‐yl)thiosemicarbazone (HL^a^), 2‐benzoylpyridine 4‐allylthiosemicarbazone (HL^b^), and their copper(II) coordination compounds. To simplify the comparison of results and make it more illustrative, the activity data are presented as diagrams for the microorganisms *Staphylococcus aureus* and *Bacillus cereus* (Figure 4). The thiosemicarbazone **HL** itself showed no activity against the tested microorganisms; however, compounds containing related structural fragments exhibited measurable effects. For comparison, copper(II) complexes with chloride, nitrate, and dichloroacetate counterions were evaluated. In the case of *S. aureus*, the copper(II) chloride complex synthesized in this study was found to be less active than the previously reported 2‐benzoylpyridine 4‐allylthiosemicarbazone complex but more active than the 2‐acetylpyridine 4‐(bicyclo [2.2.1]hept‐2‐yl)thiosemicarbazone analog, indicating that incorporation of a 4‐(bicyclo [2.2.1]hept‐2‐yl) fragment reduces antibacterial potency. For the copper(II) nitrate complexes, the structural analogs displayed comparable activity, which was consistently higher than that of the compound reported here. This suggests that merging the two structural fragments does not enhance antibacterial properties. For the copper(II) dichloroacetate complex, the MBC value was the same, while the MIC of the earlier reported compound was higher.

Similar trends were observed in the assays against *B. cereus*, where previously published copper(II) complexes generally exhibited greater or, in the case of the nitrate complex, comparable activity. Overall, these comparisons highlight the nuanced influence of structural fragments and counterions on the biological properties of copper(II)‐thiosemicarbazone systems.

## 3 Materials and Methods

### 3.1 Materials

All the reagents used were chemically pure. Metal salts: CuCl_2_·2H_2_O, Cu(NO_3_)_2_ · 3H_2_O, Cu(Cl_2_CHCOO)_2_·H_2_O, Ni(NO_3_)_2_· 6H_2_O, Fe(NO_3_)_3_·6H_2_O, Co(NO_3_)_3_·6H_2_O (Merck, Darmstadt, Germany) were used as supplied. Hydrazine hydrate, 2‐isothiocyanatobicyclo [2.2.1]heptane, 2‐benzoylpyridine, hydrochloric acid 37%, and sodium carbonate were used as received (Sigma–Aldrich, Munich, Germany).

4‐(bicyclo [2.2.1]heptan‐2‐yl)thiosemicarbazide was prepared through the condensation of 2‐isothiocyanatobicyclo [2.2.1]heptane with hydrazine hydrate, employing the procedure outlined in [31].

The NMR measurements were carried out on a Bruker DRX‐400 spectrometer with CDCl_3_ as the solvent. FTIR spectra were recorded on a Bruker ALPHA FTIR spectrophotometer at room temperature in the range of 4000–400 cm^–1^. UV–Vis absorption spectra in ethanol solutions were measured on an Agilent Cary 300 UV–Vis spectrophotometer. The elemental analysis was performed similarly to the literature procedures [32] and on the automatic Perkin Elmer 2400 elemental analyzer. The resistance of solutions of complexes in MeOH (20°C, c 0.001 M) was measured using an R‐38 rheochord bridge.

### 3.2 Synthesis

#### 3.2.1 Synthesis of 2‐benzoylpyridine 4‐(bicyclo [2.2.1]hept‐2‐yl)thiosemicarbazone (HL)

In the first stage, 4‐(bicyclo [2.2.1]hept‐2‐yl)thiosemicarbazide (0.500 g, 2.70 mmol) was dissolved in 20 mL of ethanol under continuous stirring. A solution of 2‐benzoylpyridine (0.494 g, 2.70 mmol) in 20 mL of ethanol was then added, followed by concentrated hydrochloric acid (0.274 g, 2.70 mmol). The reaction mixture was stirred and heated for 30 min. Upon cooling, a yellow solid 2‐benzoylpyridine 4‐(bicyclo [2.2.1]hept‐2‐yl)thiosemicarbazone hydrochloride precipitated. It was isolated by filtration and washed with small portions of cold ethanol.

In the second stage, the obtained hydrochloride salt was dissolved in 20 mL of ethanol under stirring and heating. The solution was neutralized using an aqueous solution of Na_2_CO_3_, and the thiosemicarbazone was extracted with chloroform.

White solid. Yield: 94%; mp 189°C‐191°C. FW: 350.48 g/mol; Anal Calc. for C_20_H_22_N_4_S: C, 68.54; H, 6.33; N, 15.99; S, 9.15; found: C, 68.43; H, 6.23; N, 15.88; S, 9.05. Main FTIR peaks (cm^−1^): ν(NH) 3368, 3151, ν(C = N) 1583, ν(C = S) 1313. λ, nm (ε, M^−1^ cm^−1^): 243 (1.36·10^4^), 275 (1.3·10^4^), 331 (2.4·10^4^). ^1^H NMR (CDCl_3_, 400 MHz) 13.55 (s, 1H, NH), 8.82 (d, 1H, NH); 7.76 (t, 1H, CH(pyridine)); 7.60 (d, 1H, CH(pyridine)); 7.45 (m, 5H, CH(phenyl)); 7.37 (t, 1H, CH(pyridine)); 7.27 (d, 1H, CH (pyridine)); 4.23 (t, 1H, CH‐N); 2.45, 2.32 (m, 2H, 2×CH (norbornyl); 2.0–1.1 (m, 8H, 4×CH_2_ (norbornyl)). ^13^C NMR (CDCl_3_, 100 MHz) 177.03 (C = S); 152.41, 142.29, 137.72, 137.15, 129.69, 129.13, 128.59, 126.10, 124.13 (C aromatic); 148.68 (C = N); 57.35, 42.32, 40.36, 35.95, 35.88, 28.16, 26.40 (C from bicyclic fragment).

#### 3.2.2 Synthesis of Coordination Compounds

##### 3.2.2.1 [Cu(L)Cl] (1)

Copper(II) chloride (0.171 g, 1 mmol) was introduced into a hot ethanolic solution (25 mL, 55°C) containing 2‐benzoylpiridine 4 ‐(bicyclo [2.2.1]hept‐2‐yl)thiosemicarbazone **HL** (0.350 g, 1 mmol). The reaction mixture was maintained under stirring at 55°C for 30 min. Upon cooling to room temperature, a green solid formed, which was collected by filtration, rinsed with cold ethanol, and subsequently dried under vacuum.

Green solid. Yield: 76%. Anal. Calc. for C_20_H_21_ClCuN_4_S (448.47 g mol^−1^): C, 53.56; H, 4.72; Cl, 7.91; Cu, 14.17; N, 12.49; S, 7.15. Found: C, 52.45; H, 4.60; Cl, 7.80; Cu, 14.05; N, 12.39; S, 7.06. Main FTIR peaks (cm^−1^): ν(NH) 3315, ν(C = N) 1614, 1583, ν(C‐S) 745. χ(CH_3_OH): 46 Ω^−1^ cm^−2^ mol^−1^. λ, nm (ε, M^−1^ cm^−1^): 260 (1.7·10^4^), 309 (2.0·10^4^), 427 (1.9·10^4^).

##### 3.2.2.2 [Cu(L)NO_3_] (2)

Copper(II) nitrate trihydrate (0.242 g, 1 mmol) was introduced into a hot ethanolic solution (25 mL, 55°C) containing 2‐benzoylpiridine 4 ‐(bicyclo [2.2.1]hept‐2‐yl)thiosemicarbazone **HL** (0.350 g, 1 mmol). The reaction mixture was maintained under stirring at 55°C for 30 min. Upon cooling to room temperature, a green solid formed, which was collected by filtration, rinsed with cold ethanol, and subsequently dried under vacuum.

Green solid. Yield: 80%. Anal. Calc. for C_20_H_21_CuN_5_O_3_S (475.02 g mol^−1^): C, 50.57; H, 4.46; Cu, 13.38; N, 14.74; S, 6.75. Found: C, 50.46; H, 4.34; Cu, 13.26; N, 14.64; S, 6.64. Main FTIR peaks (cm^−1^): ν(NH) 3311, ν(C = N) 1616, 1589, ν(C‐S) 745. χ(CH_3_OH): 80 Ω^−1^ cm^−2^ mol^−1^. λ, nm (ε, M^−1^ cm^−1^): 260 (1.7·10^4^), 309 (2.0·10^4^), 427 (1.9·10^4^).

##### 3.2.2.3 {[Cu(L)CHCl_2_COO]}_*n*_ (3)

Copper(II) dichloroacetate monohydrate (0.338 g, 1 mmol) was introduced into a hot ethanolic solution (25 mL, 55°C) containing 2‐benzoylpiridine 4 ‐(bicyclo [2.2.1]hept‐2‐yl)thiosemicarbazone **HL** (0.350 g, 1 mmol). The reaction mixture was maintained under stirring at 55°C for 30 min. Upon cooling to room temperature, a green solid formed, which was collected by filtration, rinsed with cold ethanol, and subsequently dried under vacuum.

Green solid. Yield: 72%. Anal. Calc. for C_22_H_22_Cl_2_CuN_4_O_2_S (540.95 g mol^−1^): C, 48.85; H, 4.10; Cl, 13.11; Cu, 11.75; N, 10.36; S, 5.93. Found: C, 48.74; H, 4.01; Cl, 13.11; Cu, 11.65; N, 10.24; S, 5.83. Main FTIR peaks (cm^−1^): ν(NH) 3321, ν(C = N) 1612, 1571, ν(C‐S) 746. χ(CH_3_OH): 76 Ω^−1^ cm^−2^ mol^−1^. λ, nm (ε, M^−1^ cm^−1^): 260 (1.7·10^4^), 309 (2.0·10^4^), 427 (1.9·10^4^).

##### 3.2.2.4 [Ni(HL)_2_](NO_3_)_2_ (4)

Nickel(II) nitrate hexahydrate (0.291 g, 1 mmol) was introduced into a hot ethanolic solution (25 mL, 55°C) containing 2‐benzoylpiridine 4 ‐(bicyclo [2.2.1]hept‐2‐yl)thiosemicarbazone **HL** (0.700 g, 2 mmol). The reaction mixture was maintained under stirring at 55°C for 30 min. Upon cooling to room temperature, a green solid formed, which was collected by filtration, rinsed with cold ethanol, and subsequently dried under vacuum.

Green solid. Yield: 72%. Anal. Calc. for C_40_H_44_N_10_NiO_6_S_2_ (883.66 g mol^−1^): C, 54.37; H, 5.02; N, 15.85; Ni, 6.64; S, 7.26. Found: C, 54.26; H, 4.91; N, 15.75; Ni, 6.55; S, 7.16. Main FTIR peaks (cm^−1^): ν(NH) 3361, 3154, ν(C = N) 1583, ν(C = S) 1316. χ(CH_3_OH): 134 Ω^−1^ cm^−2^ mol^−1^. λ, nm (ε, M^−1^ cm^−1^): 318 (2.7·10^4^), 423 (1.6·10^4^).

##### 3.2.2.5 [Fe(L)_2_]NO_3_ (5)

Iron(III) nitrate hexahydrate (0.350 g, 1 mmol) was introduced into a hot ethanolic solution (25 mL, 55°C) containing 2‐benzoylpiridine 4 ‐(bicyclo [2.2.1]hept‐2‐yl)thiosemicarbazone **HL** (0.700 g, 2 mmol). The reaction mixture was maintained under stirring at 55°C for 30 min. Upon cooling to room temperature, a red solid formed, which was collected by filtration, rinsed with cold ethanol, and subsequently dried under vacuum.

Red solid. Yield: 70%. Anal. Calc. for C_40_H_42_FeN_9_O_3_S_2_ (816.80 g mol^−1^): C, 58.82; H, 5.18; N, 15.43; Fe, 6.84; S, 7.85. Found: C, 58.71; H, 5.07; N, 15.32; Fe, 6.73; S, 7.73. Main FTIR peaks (cm^−1^): ν(NH) 3294, ν(C = N) 1644, 1591, ν(C‐S) 748. χ(CH_3_OH): 70 Ω^−1^ cm^−2^ mol^−1^. λ, nm (ε, M^−1^ cm^−1^): 256 (2.7·10^4^), 330 (1.7·10^4^), 380 (1.9·10^4^), 510 (0.7·10^4^).

##### 3.2.2.6 [Co(L)_2_]NO_3_ (6)

Cobalt(II) nitrate hexahydrate (0.350 g, 1 mmol) was introduced into a hot ethanolic solution (25 mL, 55°C) containing 2‐benzoylpiridine 4‐(bicyclo [2.2.1]hept‐2‐yl)thiosemicarbazone **HL** (0.700 g, 2 mmol). The reaction mixture was maintained under stirring at 55°C for 30 min. Upon cooling to room temperature, a red solid formed, which was collected by filtration, rinsed with cold ethanol, and subsequently dried under vacuum.

**FIGURE 5:**
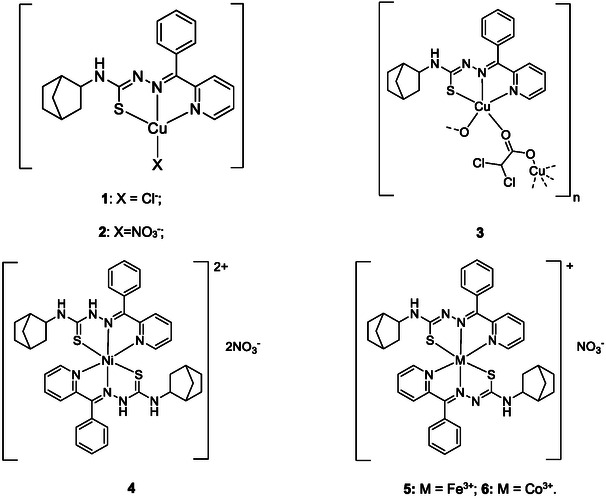
Proposed chemical structures of complexes **1**–**6**.

Red solid. Yield: 71%. Anal. Calc. for C_40_H_42_CoN_9_O_3_S_2_ (819.88 g mol^−1^): C, 58.60; H, 5.16; Co, 7.19; N, 15.38; S, 7.82. Found: C, 58.49; H, 5.06; Co, 7.08; N, 15.27; S, 7.71. Main FTIR peaks (cm^−1^): ν(NH) 3311, ν(C = N) 1591, 1559, ν(C‐S) 743. χ(CH_3_OH): 70 Ω^−1^ cm^−2^ mol^−1^. λ, nm (ε, M^−1^ cm^−1^): 319 (3.2·10^4^), 385 (3.0·10^4^), 435 (1.6·10^4^). The proposed chemical structures of complexes 1–6 are given in Figure 5.

### 3.3 X‐Ray Crystallography

The X‐ray diffraction data for the crystals of **HL** and complex **3** were collected on the Xcalibur E diffractometer equipped with a charge‐coupled device (CCD) area detector and a graphite monochromator utilizing MoKα radiation (0.71073 Å) at room temperature (293 K). Data collection, cell refinement, and data reduction were performed using the CrysAlis PRO CCD (Oxford Diffraction) [33]. The structure solution and refinement were carried out using the SHELXS97 and SHELXL2014 program suites [34, 35]. The structures were solved by direct methods and refined by full‐matrix least‐squares on *F* ^2^ with anisotropic displacement parameters for all non‐hydrogen atoms. The C‐bound H–atoms were positioned geometrically and treated as riding atoms using SHELXL default parameters with U_iso_(H) = 1.2U_eq_(C) and U_iso_(H) = 1.5U_eq_(CH_3_). The (bicyclo [2.2.1]hept‐2‐yl) fragment in **HL** is disordered over two positions with an equal probability.

The X‐ray data and structure refinement details are summarized in Table 1. Bond lengths (Å) and angles (deg) in the coordination metal environment in **3** are summarized in Table S1, while hydrogen bonding parameters for **HL** and complex **3** can be found in Table S2. The figures were produced using MERCURY software (version 2025.2.0, Build 454 209; Cambridge Crystallographic Data Centre, Cambridge, UK, 2025).

### 3.4 Antibacterial and Antifungal Activity

The antibacterial and antifungal potential of **HL** and its coordination compounds **1**–**6** was evaluated against reference strains of *Staphylococcus aureus* (ATCC 25 923), *Bacillus cereus* (ATCC 11 778), *Acinetobacter baumannii* (BAA‐747), *Escherichia coli* (ATCC 25 922), and *Candida albicans* (ATCC 10 231). Minimum inhibitory concentrations (MIC, μg mL^−1^), minimum bactericidal concentrations (MBC, μg mL^−1^), and minimum fungicidal concentrations (MFC, μg mL^−1^) were established using the broth microdilution method. Stock solutions of the investigated compounds were prepared in DMSO at 10 mg mL^−1^, and subsequent dilutions were carried out with 2% peptone broth. Furacillinum [36, 37] served as the antibacterial reference drug, while Nystatin [36] and Fluconazole [38] were employed as standard antifungal agents.

## 4 Conclusion

The new copper(II), nickel(II), cobalt(III), and iron(III) complexes with 2‐benzoylpyridine 4‐(bicyclo [2.2.1]hept‐2‐yl)thiosemicarbazone (**HL**) were obtained. The antibacterial and antifungal activities were studied for **HL** and its complexes **1**‐**6**. The results showed that coordination of the thiosemicarbazone **HL** to metal atoms led to an increase in antibacterial activity, whereas in the case of antifungal activity, the obtained complexes did not exceed the activity of the thiosemicarbazone **HL**. Most of the complexes demonstrated better results than the reference drugs Furacillinum, Nystatin, and Fluconazole. The activity of the complexes is influenced by the counterion or the metal atom, which confirms that modifying the structure can affect the biological activity of the compounds.

## Supporting Information

Additional supporting information can be found online in the Supporting Information section. NMR spectra of **HL**, FTIR spectra of studied compounds, tables with bond lengths and angles in coordination metal environment in **3**, and hydrogen bond distances and angles in **HL** and **3** are given in supporting information. Deposition Numbers 2493073 (for **HL**), 2493074 (for **3**) contain the supplementary crystallographic data for this paper. These data are provided free of charge by the joint Cambridge Crystallographic Data Centre and Fachinformationszentrum Karlsruhe Access Structures service. **Supporting Fig. S1**: ^1^H NMR (400 MHz, CDCl_3_) spectrum of 2‐benzoylpyridine *N*4‐(bicyclo[2.2.1]hept‐2‐yl)thiosemicarbazone (**HL**). **Supporting Fig. S2**: ^13^C NMR (400 MHz, CDCl_3_) spectrum of 2‐benzoylpyridine *N*4‐(bicyclo[2.2.1]hept‐2‐yl)thiosemicarbazone (**HL**). **Supporting Fig. S3**: FTIR spectrum of **HL. Supporting Fig. S4**: FTIR spectrum of **1. Supporting Fig. S5**: FTIR spectrum of **2. Supporting Fig. S6**: FTIR spectrum of **3. Supporting Fig. S7**: FTIR spectrum of **4. Supporting Fig. S8**: FTIR spectrum of **5. Supporting Fig. S9**: FTIR spectrum of **6. Supporting Table S1**: Bond Lengths (Å) and Angles (deg) in Coordination Metal Environment in **3**. **Supporting Table S2**: Hydrogen bond distances (Å) and angles (°) in **HL** and **3**.

## Funding

Agenţia Naţională pentru Cercetare şi Dezvoltare (24.80012.5007.14TC); Institutional Project (010602, 011202).

## Supporting information

Supplementary Material
