# Supplementary material for: From Design to Bioactivity: 2‐Benzoylpyridine 4‐(bicyclo[2.2.1]hept‐2‐yl)thiosemicarbazone and Its 3d Metal Coordination Compounds
Source: ChemistryOpen. 2026 Mar 29;15(4):e202500590. doi: 10.1002/open.202500590 (PMC13140696; doi:10.1002/open.202500590)
Supplement: Supplementary file 1 — Supplementary Material [file OPEN-15-e202500590-s001.pdf]

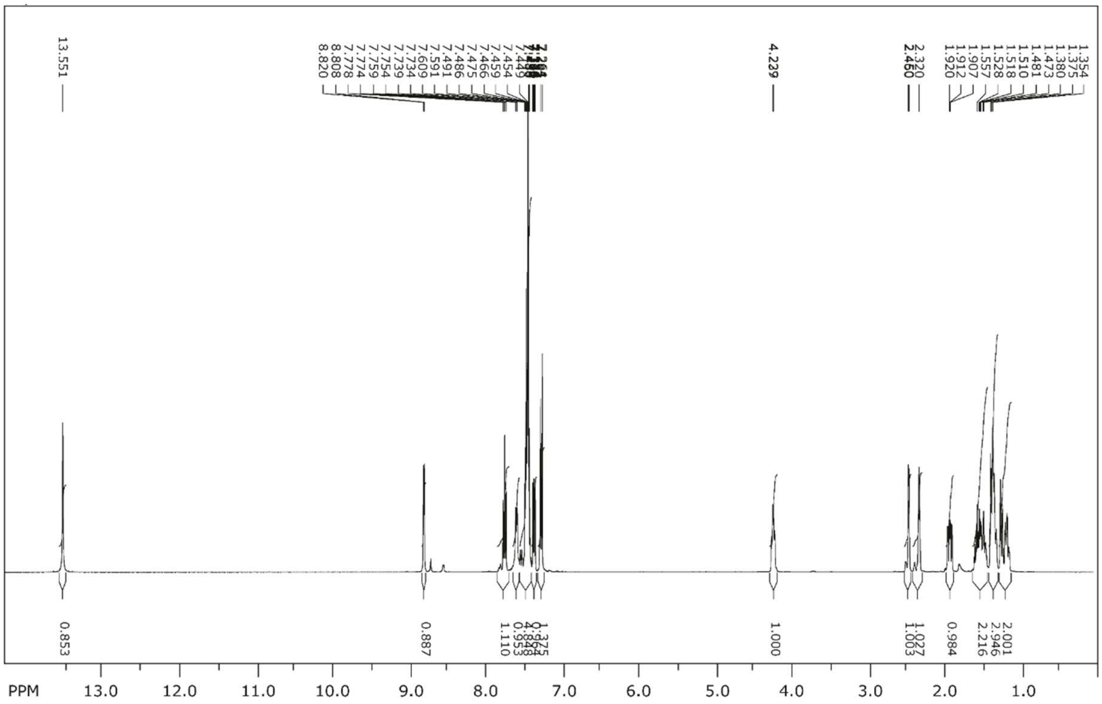

Figure S1. ^{1}H NMR (400 MHz, $CDCl_3$) spectrum of 2-benzoylpyridine *N*4-(bicyclo[2.2.1]hept-2-yl)thiosemicarbazone (**HL**)

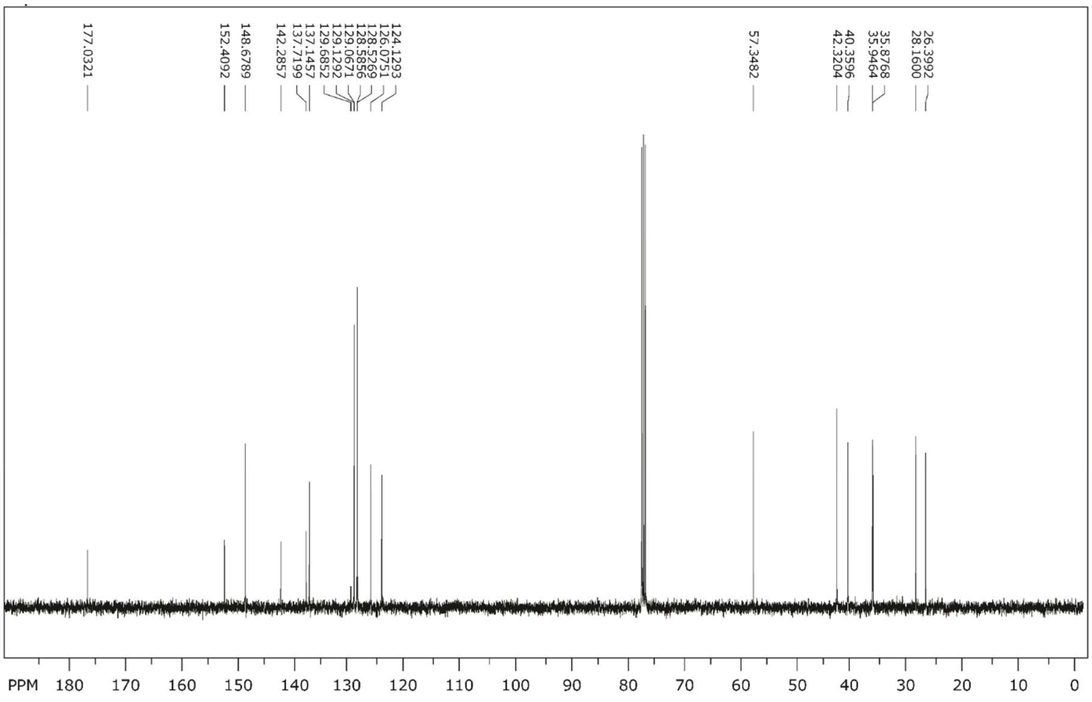

Figure S2. ^{13}C NMR (400 MHz, $CDCl_3$) spectrum of 2-benzoylpyridine *N*4-(bicyclo[2.2.1]hept-2-yl)thiosemicarbazone (**HL**)

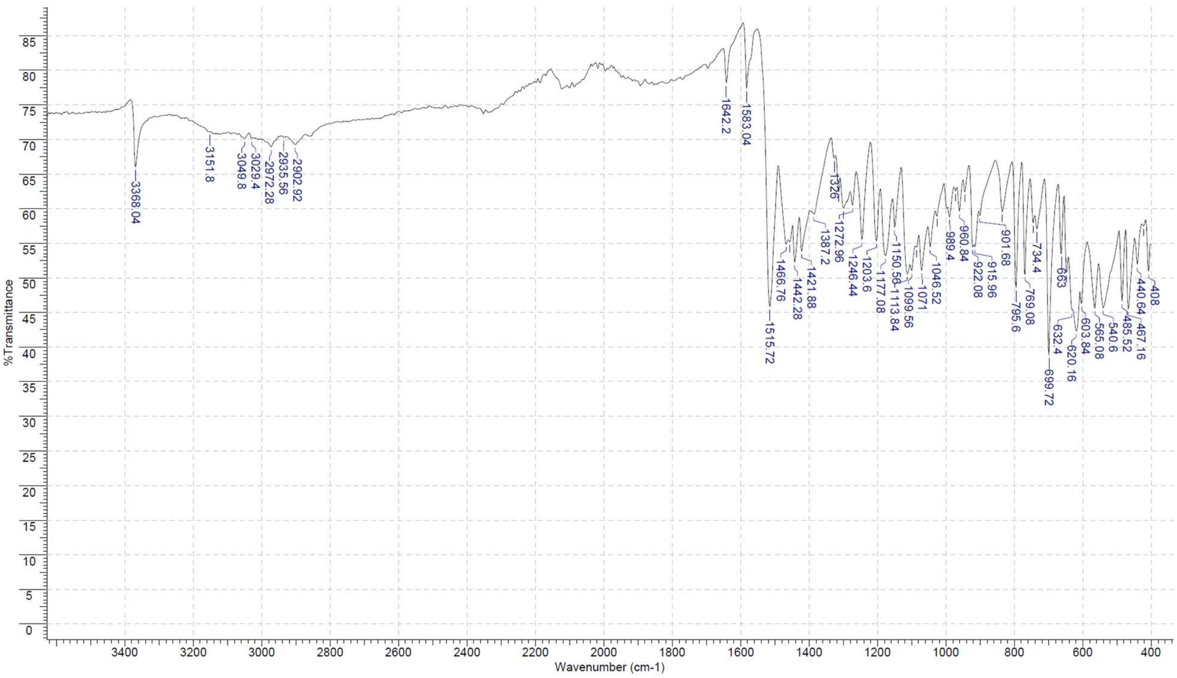

Figure S3. FTIR spectrum of **HL**

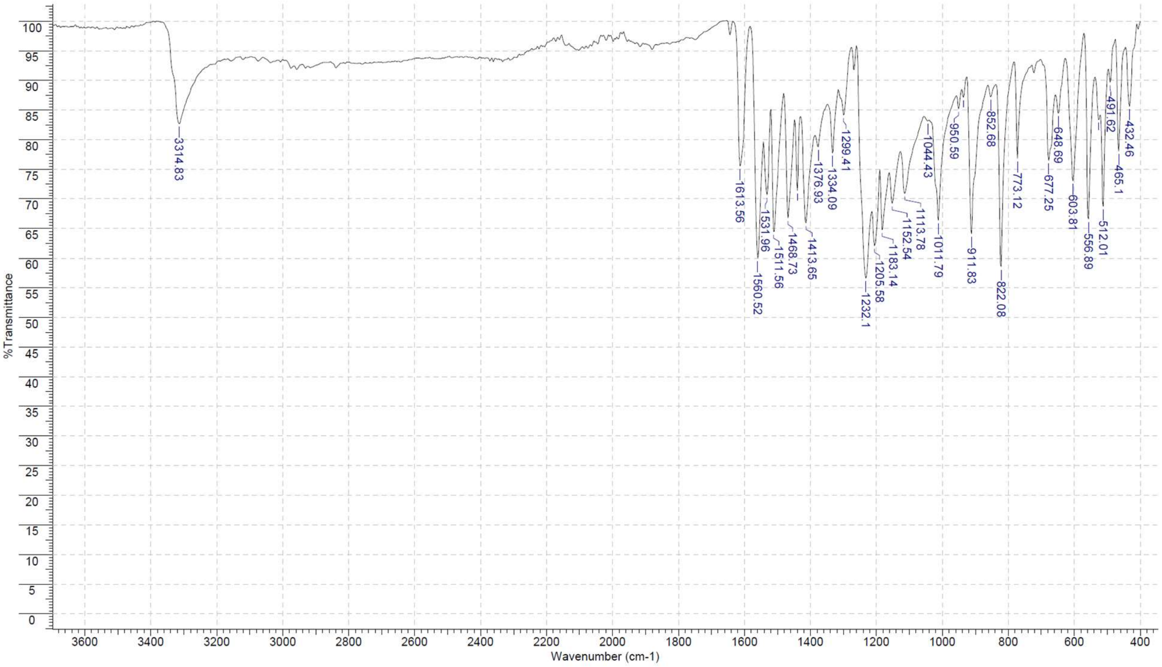

Figure S4. FTIR spectrum of **1**

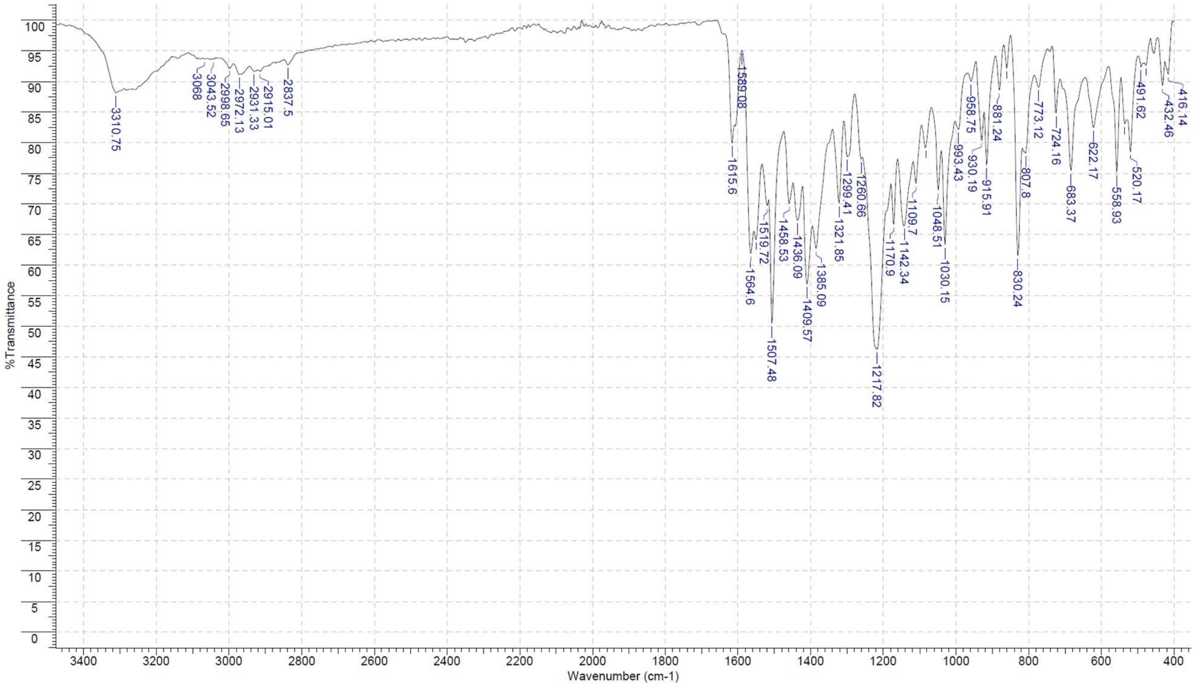

Figure S5. FTIR spectrum of **2**

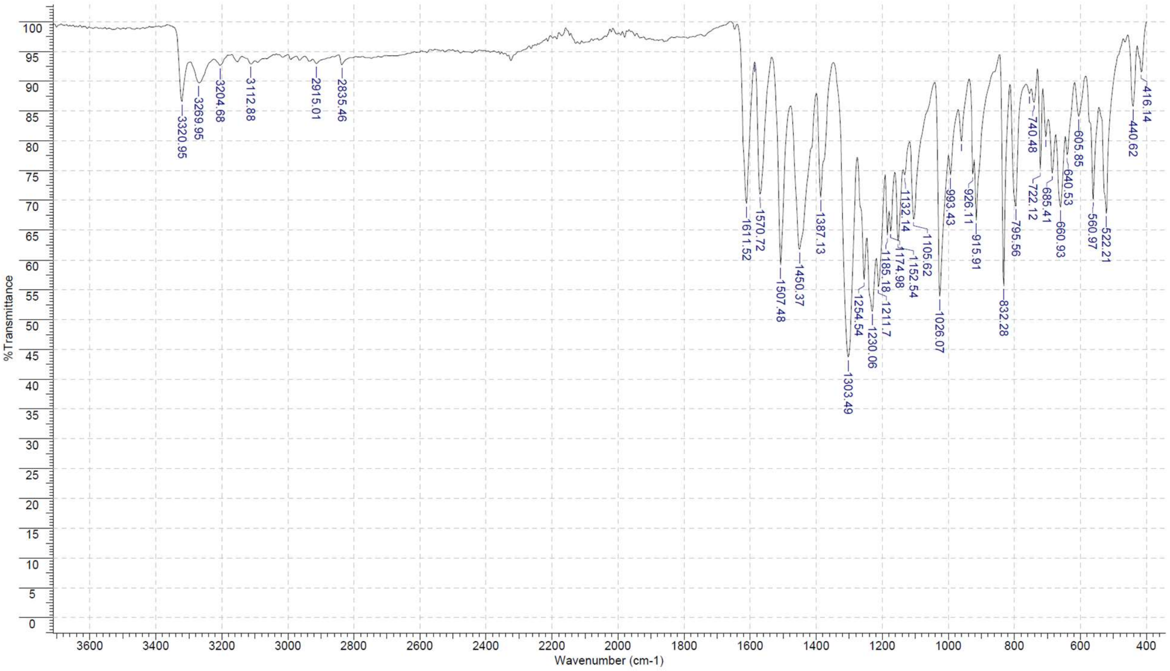

Figure S6. FTIR spectrum of **3**

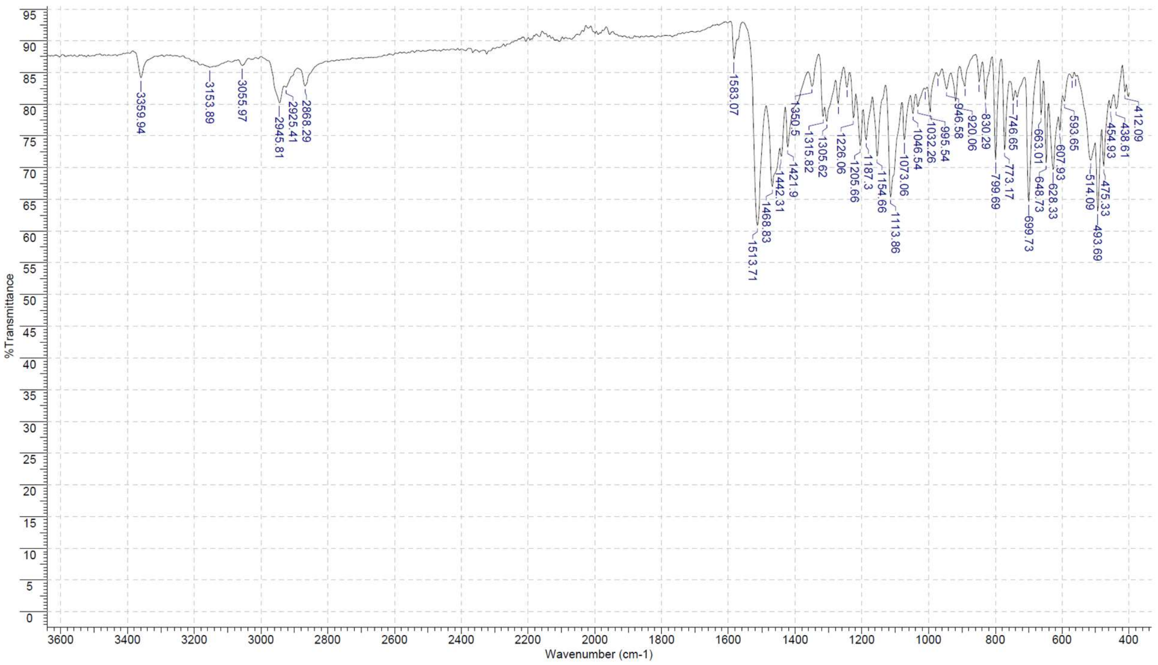

Figure S7. FTIR spectrum of **4**

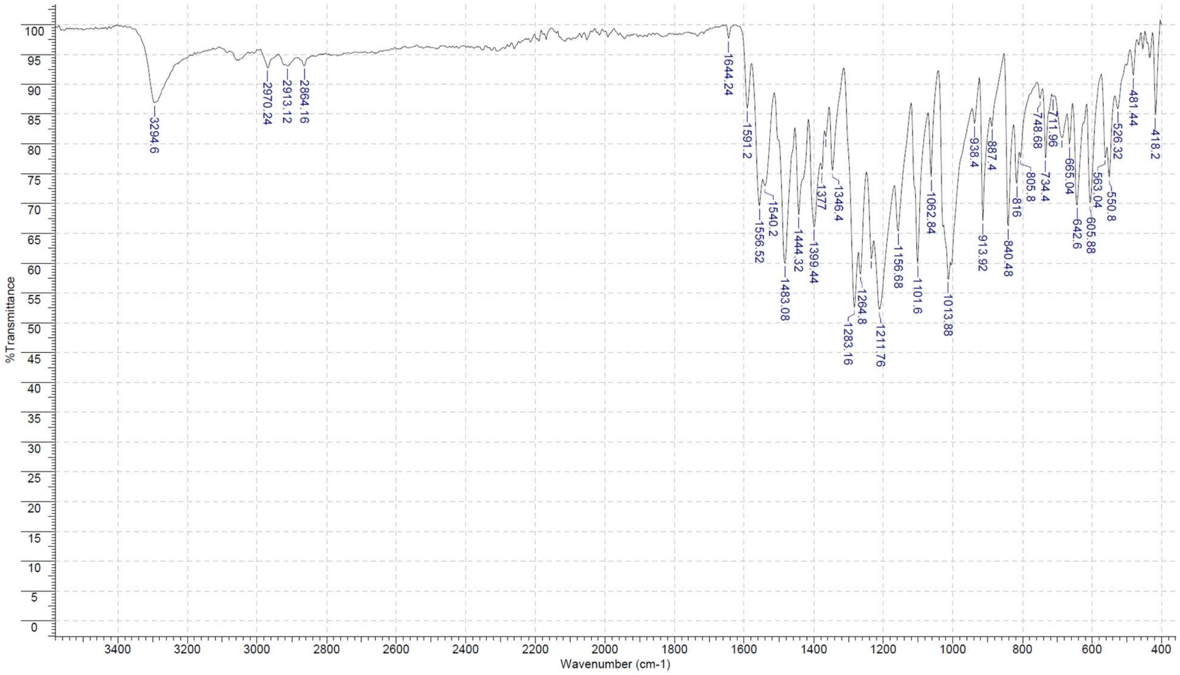

Figure S8. FTIR spectrum of **5**

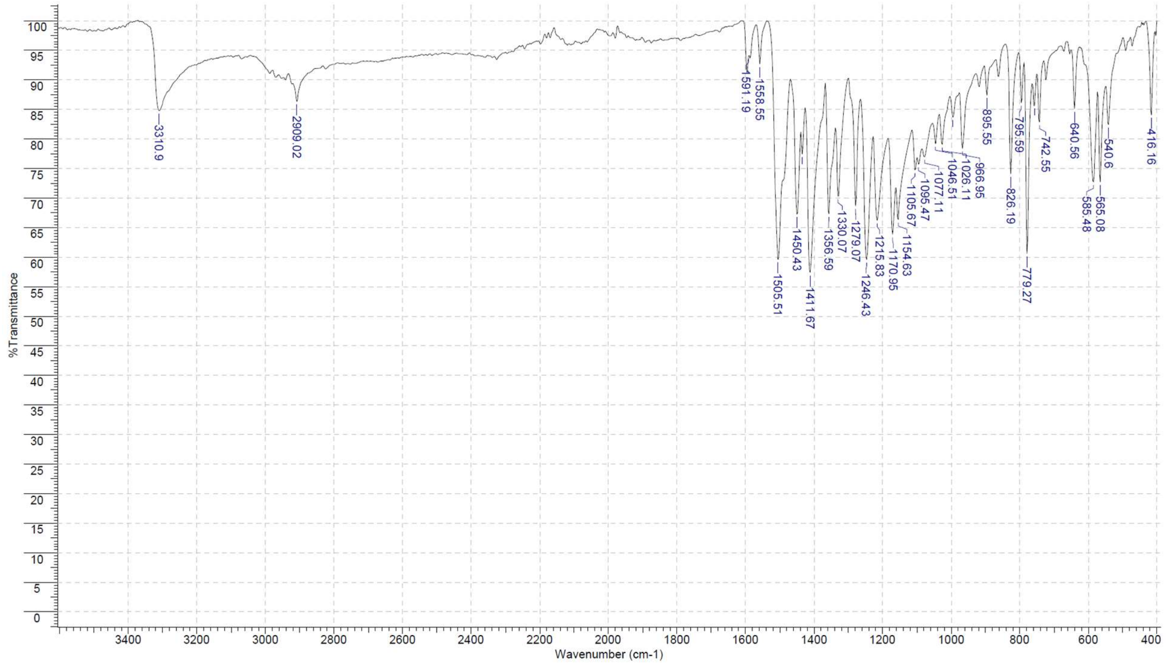

Figure S9. FTIR spectrum of **6**

Table S1. Bond Lengths (Å) and Angles (deg) in Coordination Metal Environment in **3**.

Bond lengths (Å)			
Cu(1)-N(2)	1.958(4)	Cu(1)-S(1)	2.268(2)
Cu(1)-O(2)^{I}	1.967(3)	Cu(1)-O(1)	2.320(4)
Cu(1)-N(1)	2.009(5)		
Bond angles (°)			
N(2)-Cu(1)-O(2)^{I}	166.6(2)	N(1)-Cu(1)-S(1)	164.5(2)
N(2)-Cu(1)-N(1)	80.5(2)	N(2)-Cu(1)-O(1)	99.4(2)
O(2)^{I}-Cu(1)-N(1)	95.7(2)	O(2)^{I}-Cu(1)-O(1)	92.9(2)

N(2)-Cu(1)-S(1)	84.1(2)	N(1)-Cu(1)-O(1)	84.4(2)
O(2)^{I}-Cu(1)-S(1)	98.81(14)	S(1)-Cu(1)-O(1)	100.33(13)

Symmetry transformations used to generate equivalent atoms: ^{I} *x*, -*y*+1/2, *z*-1/2

Table S2. Hydrogen bond distances (Å) and angles (°) in **HL** and **3**.

D–H···A	*d*(H···A)	*d*(D···A)	∠(DHA)	Symmetry transformations for acceptor
**HL**				
N(3)–H(3N)···N(1)	2.04	2.691(3)	131.8	*x*, *y*, *z*
C(6)-H(6)···S(1)	2.94	3.719(3)	142.4	-*x*-1, -*y*+1, -*z*+1
**3**				
C(22)–H(22)···O(2)	2.23	3.158(8)	156.5	*x, -y*+1/2*, z*-1/2
N(4)–H(4N)···S(1)	2.68	3.530(5)	174(5)	*-x*+1*, -y, -z*+1
